# Plasma IP-10, apoptotic and angiogenic factors associated with fatal cerebral malaria in India

**DOI:** 10.1186/1475-2875-7-83

**Published:** 2008-05-19

**Authors:** Vidhan Jain, Henry B Armah, Jon E Tongren, Renée M Ned, Nana O Wilson, Sara Crawford, Pradeep K Joel, Mrigendra P Singh, Avinash C Nagpal, AP Dash, Venkatachalam Udhayakumar, Neeru Singh, Jonathan K Stiles

**Affiliations:** 1 National Institute of Malaria Research (ICMR), Jabalpur, India; 2 Department of Microbiology, Biochemistry and Immunology, Morehouse School of Medicine, Atlanta, Georgia, USA; 3 Malaria Branch, Division of Parasitic Diseases, National Center for Zoonotic, Vector-Borne and Enteric Diseases, Coordinating Center for Infectious Diseases, Centers for Disease Control and Prevention, Atlanta, Georgia, USA; 4 Nethaji Subash Chandra Bose Hospital, Jabalpur, Madhyapradesh, India

## Abstract

**Background:**

*Plasmodium falciparum *in a subset of patients can lead to cerebral malaria (CM), a major contributor to malaria-associated mortality. Despite treatment, CM mortality can be as high as 30%, while 10% of survivors of the disease may experience short- and long-term neurological complications. The pathogenesis of CM is mediated by alterations in cytokine and chemokine homeostasis, inflammation as well as vascular injury and repair processes although their roles are not fully understood. The hypothesis for this study is that CM-induced changes in inflammatory, apoptotic and angiogenic factors mediate severity of CM and that their identification will enable development of new prognostic markers and adjunctive therapies for preventing CM mortalities.

**Methods:**

Plasma samples (133) were obtained from healthy controls (HC, 25), mild malaria (MM, 48), cerebral malaria survivors (CMS, 48), and cerebral malaria non-survivors (CMNS, 12) at admission to the hospital in Jabalpur, India. Plasma levels of 30 biomarkers ((IL-1β, IL-1ra, IL-2, IL-4, IL-5, IL-6, IL-8, IL-9, IL-10, IL-12 (p70), IL-13, IL-15, IL-17, Eotaxin, FGF basic protein, G-CSF, GM-CSF, IFN-γ, IP-10, MCP-1 (MCAF), MIP-1α, MIP-1β, RANTES, TNF-α, Fas-ligand (Fas-L), soluble Fas (sFas), soluble TNF receptor 1 (sTNF-R1) and soluble TNF receptor 2 (sTNFR-2), PDGF bb and VEGF)) were simultaneously measured in an initial subset of ten samples from each group. Only those biomarkers which showed significant differences in the pilot analysis were chosen for testing on all remaining samples. The results were then compared between the four groups to determine their role in CM severity.

**Results:**

IP-10, sTNF-R2 and sFas were independently associated with increased risk of CM associated mortality. CMNS patients had a significantly lower level of the neuroprotective factor VEGF when compared to other groups (P < 0.0045). The ratios of VEGF to IP-10, sTNF-R2, and sFas distinguished CM survivors from non survivors (P < 0.0001).

**Conclusion:**

The results suggest that plasma levels of IP-10, sTNF-R2 and sFas may be potential biomarkers of CM severity and mortality. VEGF was found to be protective against CM associated mortality and may be considered for adjunctive therapy to improve the treatment outcome in CM patients.

## Background

*Plasmodium falciparum *in a subset of patients can lead to a diffuse encephalopathy known as cerebral malaria (CM), a major contributor to malaria-associated mortality [1]. Despite treatment, mortality in CM can be as high as 30% while 10% of survivors of the disease may experience short- and long-term neurological complications [2]. *Plasmodium falciparum *infection and CM have re-emerged in India, causing considerable morbidity and mortality [3].

Fatal CM is a multi-factorial neurological syndrome involving reversible vascular occlusion in the presence of sequestered parasitized erythrocytes (pRBCs) [4], leukocytes [4,5], inflammation [6], apoptosis [6-9], platelets [10] and angiogenic failures [11]. Although sequestration of parasites may occur in any vascular bed, the phenomenon of mechanical obstruction within the brain appears to be the result of the adherence of pRBCs to other pRBCs, RBCs, and the endothelium [5,12,13]. Parasite sequestration alone cannot account for fatal CM pathogenesis as there is evidence that survivors of the disease have the same degree of sequestration during comas as those succumbing to disease [14]. It is evident that a combination of parasite and host immune factors play an important role in the pathogenesis of CM [15].

Clinical studies and murine models support a role for pro-inflammatory (Th1) and anti-inflammatory (Th2) cytokines and chemokines in the pathogenesis of CM [16,17]. Although pro-inflammatory cytokines play a dominant role in parasite destruction and conferring protection [18], they are also associated with CM pathogenesis. Increased plasma levels of TNF-α up regulates the expression of adhesion molecules, thereby exacerbating parasite sequestration [19], and correlate with severe disease [20]. Plasma levels of soluble TNF receptor 1 (sTNF-R1) and 2 (sTNF-R2), which act as binding proteins for TNF-α, have been shown to increase in Malawian children [21] and in post-mortem CSF and serum in Ghanaian CM non-survivors [22] when compared with levels in convalescent children and in healthy controls. Severe malaria has also been associated with increased production of IFN-γ and IL-1β [4] and decreased production of anti-inflammatory cytokines, notably IL-10 and TGF-β [23].

Chemokines of the C-C or β subfamily, including macrophage inflammatory proteins 1 (MIP-1α), MIP-1β, and Regulated on Activation Normal T cell Expressed and Secreted (RANTES/CCL5) modulate many infectious and inflammatory diseases, including malaria. Animal and post-mortem human studies indicate that expression of RANTES/CCL5, a chemo-attractant for T cells and monocytes, and its corresponding receptors CCR3 and CCR5 are elevated in brain tissues of C57BL/6 mice infected with *Plasmodium berghei *ANKA and in post-mortem tissues of CM patients [24,25]. Although recent observations in Uganda have shown a significant reduction in RANTES in peripheral blood of severe malaria patients (SM) [26], MIP-1α and MIP-1β serum levels were elevated [27]. Interferon inducible protein 10 (IP-10), a member of the CXC or α subfamily, is induced in response to IFN-γ, which attracts activated Th1 cells [28]. IP-10 levels are elevated in cultured intervillous blood mononuclear cells isolated from placenta's infected with malaria [29,30], and in post-mortem CSF and sera in non survivors of CM [22].

Angiogenic factors, long implicated in ischaemia (stroke) prognoses [31], may also be involved in CM-induced petechial haemorrhages and CM-associated brain endothelium/blood brain barrier (BBB) dysfunction [32]. Vascular endothelial growth factor (VEGF) stimulates endothelial cell growth, migration, and enhances vascular permeability. In a post-mortem immunohistological evaluation of CM patients, VEGF levels (VEGF+ astrocytes) were higher in CM patients compared with controls [11]. Platelets, which accumulate with pRBCs in the brain microvasculature in CM patients, are also implicated in CM pathology [10,33]. Platelet derived growth factor (PDGF) stimulates vascular growth and induces regeneration of damaged axons and neuronal growth after ischemia [31]. PDGFbb levels were lower than that of controls in post-mortem CSF and sera in non-survivors of CM and were predictive of CM associated mortality [22].

The goal of this study was to test the hypothesis that CM-induced changes in inflammatory, apoptotic and angiogenic factors mediate severity of CM in India. By evaluating these factors simultaneously using high throughput multiplex immunoassay techniques, characterization of these factors is accelerated and identification of new targets of adjunctive therapies for preventing CM mortalities is enabled. A prospective study was conducted in Jabalpur, India to assess the levels of peripheral blood pro-inflammatory, apoptotic and angiogenic factors associated with CM in healthy controls (HC), mild malaria (MM) patients, CM survivors (CMS), and CM non-survivors (CMNS). The levels of immunologically-relevant mediators of malaria were evaluated to determine their role in the progression of CM immunopathology in India. The findings suggest critical roles of IP-10, apoptotic factors and angiogenic factors in CM-associated mortality.

## Methods

### Study site

The study was conducted in the Jabalpur province, Madhya Pradesh, India, which is a malaria-endemic region that accounts for 23% of all malaria cases in the state [34]. The study was carried out at two sites: Nethaji Subash Chandra Bose (NSCB) Hospital (a regional referral hospital) in Jabalpur and Civil Hospital (a primary hospital) in Maihar, Satna District. Both *Plasmodium vivax *and *P. falciparum *are prevalent in this area, and *P. falciparum *transmission occurs primarily during the monsoon and post-monsoon seasons (July – January). Previous studies revealed that malaria is present in all age groups, with the highest prevalence occurring in children between 8–14 years of age [35].

### Study subjects

Patient recruitment was initiated in October 2004 and ended in December 2006. All subjects were enrolled after obtaining informed consent from patients and guardians of patients with unarousable coma. Informed consent and human subject research guidelines of the National Institutes of Health (NIH, USA), and the ethical committees of the Morehouse School of Medicine (USA), the National Institute of Malaria Research (India), and the Centers for Disease Control and Prevention (CDC) in the United States were followed. The total numbers of subjects included in this study are summarized in Table 1. The primary aim was to compare the immunologic profiles between MM, CMS and CMNS. Healthy controls (HC) were included as an additional control group to estimate the steady state levels of various biomarkers in this study. Thus, the HC numbers were not equal to that of the malaria groups. Pregnant women and patients with other manifestation of severe disease such as respiratory distress without CM and non-CM related coma, were excluded from the study. Data relating to age, sex, and level of parasitaemia as well as complications such as seizure, renal failure, acute respiratory failure, impaired hepatic function, haematuria/spontaneous bleeding/DIC, and psychiatric disorder, were obtained from medical records. It was not possible to include data on non malaria deaths (i.e., patients dying of severe neurological disease or of other non-cerebral disease) to relate specificity of mortalities to malaria alone. However, recent analyses of CSF and serum in fatal Ghanaian CM cases revealed that the observations were not part of a generalized fatal cascade in human disease [22].

**Table 1:** Clinical characteristics of the study participants

	CATEGORY			
	
CHARACTERISTIC	HC	MM	CMS	CMNS
Total number of subjects	25	48	48	12
Adults (≥ 18 yr)	20	33	32	7
Children (< 18 yr)	5	15	16	5
No of males	18	28	34	6
No of females	7	20	14	6
Median age (yr)	22 ± 14	19 ± 14	25 ± 19	20 ± 16
Mean Glasgow Coma Score	14	14	6.7 ± 2.3	5.08 ± 2.2
Seizures	0	0	16	8
Geomean parasitaemia (pRBC/mm^3^)	0	1594 ± 426.6	4166 ± 650.9	1336 ± 386.2
Geomean haemoglobin (g/dL)	10.1 ± 0.40	9.17 ± 0.38	7.94 ± 0.37	6.82 ± 0.73
Tribal population	4	4	12	4
Non-tribal population	21	44	36	8
Renal failure	0	0	7	2
Geomean duration of admission (days)	N/A	3.5	6.4	1.8

Clinical study characteristics of the study participants in Jabalpur, India. HC – healthy controls, MM – mild malaria, CMS – cerebral malaria survivors, CMNS – cerebral malaria non-survivors. Relevant clinical data and information (such as duration of coma and seizures) were recorded for each patient from physician's records. Venous blood samples from children (2 – 5 ml) and adults (10 ml) were collected soon after enrolment into the study at the hospital from HC, MM, CMS, and CMNS groups prior to commencement of anti-malarial treatment or transfusions. Plasma was separated after centrifugation in Becton-Dickinson cell preparation tubes and aliquoted and frozen at -80°C for long-term storage.

### Enrollment criteria

#### Cerebral malaria

To be considered a case of CM, a patient had to fulfill the World Health Organization's definition of severe CM [36], have a Glasgow coma score of ≤8; have a *P. falciparum *parasitaemia, and have no other clinically evident cause of impaired consciousness [37]. CMS and CMNS remained as two separate groups. Non survivors (CMNS) were enrolled if they died within three days of admission.

#### Mild malaria

Patients who had fever with *P. falciparum *parasitaemia of <25,000 parasites/μl of blood (detected microscopically from blood smears) and no evidence of impaired consciousness, seizures, and no past history of mental illness, meningitis, or accidental head injury were included.

#### Healthy control

Relatives of patients (15) in the hospital and members of the community (10) who did not have malaria or other febrile illness were included after clinical evaluation.

Relevant clinical data and information (such as duration of coma and seizures) were recorded for each patient from physician's records [38]. Venous blood samples from children (2–5 ml) and adults (10 ml) were collected soon after enrolment into the study at the hospital from HC, MM, CMS, and CMNS groups prior to commencement of anti-malarial treatment or transfusions. Plasma was separated after centrifugation in Becton-Dickinson cell preparation tubes (catalogue #362753, BD Pharmingen, Franklin Lakes, NJ, USA), aliquoted and frozen at -80°C for long-term storage.

### Multiplexed microsphere cytokine immunoassay

Plasma (50 μL) from HC, MM, CMS and CMNS were evaluated simultaneously for 26 circulating cytokines ((IL-1β, IL-1ra, IL-2, IL-4, IL-5, IL-6, IL-8, IL-9, IL-10, IL-12 (p70), IL-13, IL-15, IL-17, Eotaxin, FGF basic protein, G-CSF, GM-CSF, IFN-γ, IP-10, MCP-1 (MCAF), MIP-1α, MIP-1β, PDGF bb, RANTES, TNF-α, and VEGF)) using a multiplex bead-based cytokine immunoassay (MMA) coupled with the Luminex™ system (Austin, TX) and human-specific bead sets (BioRad, San Diego, CA), according to the manufacturer's instructions. The results were interpolated from five-parameter-fit standard curves generated using the relevant recombinant human proteins (BioRad). Samples were tested at a 1:4 dilution.

### Enzyme-linked immunosorbent assay (ELISA)

Four (4) biomarkers, Fas-ligand (Fas-L), soluble Fas (sFas), soluble TNF receptor 1 (sTNF-R1) and soluble TNF receptor 2 (sTNFR-2), were measured by standard cytokine ELISA, using human-specific primary and secondary antibodies (Biosource, R&D, and BD Pharmingen, San Diego, CA). Results were interpolated from five-parameter-fit standard curves generated with appropriate recombinant human biomarkers (BD Pharmingen). Samples were tested at a 1:4 dilution.

### Statistical analysis

Demographic variables were compared across the four study groups using analysis of variance (for continuous variables) and chi-squared testing (for categorical variables). Non-parametric tests (Mann-Whitney rank sum tests) were used for individual cytokine analysis between groups, while multivariate analyses (Least Squares with Bonferonni correction) determined biomarker significance between groups after modeling and controlling for covariates (age, sex, and parasitaemia). Correlations between cytokine levels were assessed by Spearman's rank correlation. Box plots of biomarker concentrations outline the 25^th ^and 75^th ^percentiles, with bars representing the 10^th ^and 90^th ^percentiles. Values outside the 10^th ^and 90^th ^percentiles are plotted as points. Statistical significance (*) in CM cases were set at a two-tailed P < 0.05. Multinomial logistic regression analysis was used to identify biomarkers independently associated with disease severity. Odds ratio estimates produced from this analysis with healthy controls as the reference group were used to establish the presence of a trend of increasing disease severity for increasing biomarker levels (risk factors), or decreasing disease severity for increasing biomarker levels (protective factors). STATA™ (College Station, TX, USA) and SAS™ (Cary, NC, USA) were used to calculate statistics and plot graphs.

## Results

### Patient characteristics

A total of 133 subjects were included in this study, and their characteristics are described in Table 1. The main complications in CM patients but absent in HC and MM were seizure (CMS 33.3%, CMNS 66.67%), renal failure (CMS 14.58%, CMNS 16.7%), acute respiratory failure (CMS 25%, CMNS 50%), impaired hepatic function (CMS 18.7%, CMNS 8.3%) haematuria/spontaneous bleeding/DIC (4.51%) and psychiatric disorder (2.25%). Age, sex, and level of parasitaemia did not affect prediction of the severity of malaria among the MM, CMS and CMNS groups (Table 1). Anaemia was present in all study groups, but the haemoglobin levels were significantly lower in the CMS (p < 0.009) and CMNS (p < 0.003) patients as compared to HC. Haemoglobin levels were significantly different between MM and CMNS (p < 0.043) but not between MM and CMS groups. CM mortality rate was 9%, mostly among subjects with multi-organ failure. Adults had higher mortality rates due to the presence of multiple complications (RR-1.88, 95% CI 1.01–3.48).

### Biomarker levels in patient groups

**Figure 1:**
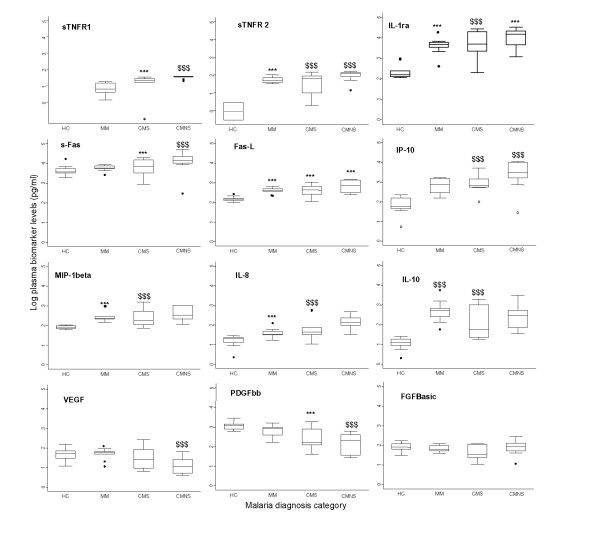
Log plasma biomarker levels and disease severity. Log median levels (pg/ml) of twelve (12) biomarkers [sTNF-R1, sTNF-R2 IL-1ra, sFas, Fas-L, IP-10, MIP-1β, IL-8, IL-10, VEGF, PDGFbb and FGF basic protein]. With the exception of sFas, Fas-L, sTNF-R1, and sTNF-R2 that were measured by ELISA, all other biomarkers were quantified via Luminex™ from blood samples collected from patients at time of admission. HC – healthy controls, MM – mild malaria, CMS – cerebral malaria survivors, CMNS – cerebral malaria non-survivors. Box plots represent medians and 25^th ^and 75^th ^percentiles. Bars mark the 10^th ^and 90^th ^percentiles, and outliers are plotted as points. HC versus MM, CMS, and CMNS: *** p < 0.05, $$$ p < 0.0045 bonferroni adjustment (alpha = 0.05/12)

**Figure 2:**
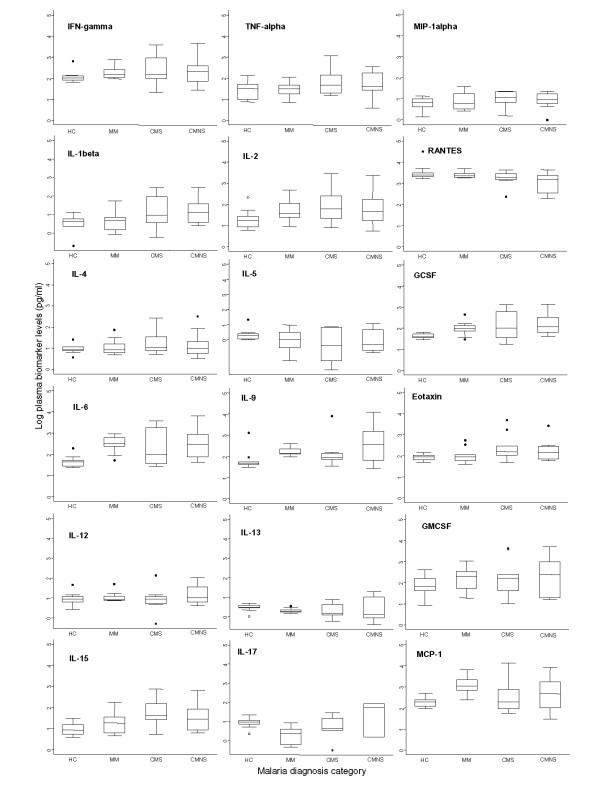
Log plasma biomarker levels and disease severity. Log median levels (pg/ml) of eighteen (18) biomarkers [IFN-γ, TNF-α, MIP-1α, IL-1β, IL-2, RANTES, IL-4, IL-5, G-CSF, IL-6, IL-9, Eotaxin, IL-12 (p70), IL-13, GM-CSF, IL-15, IL-17 and MCP-1 (MCAF)]. Biomarkers were quantified via Luminex™ from blood samples collected from patients at time of admission. HC – healthy controls, MM – mild malaria, CMS – cerebral malaria survivors, CMNS – cerebral malaria non-survivors. Box plots represent medians and 25^th ^and 75^th ^percentiles. Bars mark the 10^th ^and 90^th ^percentiles, and outliers are plotted as points. HC versus MM, CMS, and CMNS: *** p < 0.05, $$$ p < 0.0045 bonferroni adjustment (alpha = 0.05/18)

In a pilot test, ten plasma samples from each of the four disease groups (HC, MM, CMS, and CMNS) were selected and assayed for 30 biomarkers. Median biomarker levels (box plots) of the 30 biomarkers are presented in Figures 1 and 2. Many biomarkers demonstrated marginal increases, while some decreased, with increasing disease severity. Pairwise comparisons were used to determine levels of significance between disease status after controlling for age, sex and parasitaemia. Median levels of 11 biomarkers (IL-1ra, IL-8, IL-10, sTNF-R1, sTNF-R2, VEGF, IP-10, MIP-1β, PDGFbb, sFas, and Fas-L) showed significant differences between comparison groups. These biomarkers can be grouped into four major categories: cytokine receptors (sTNFR1, sTNFR2); cytokines and chemokine (Il-1ra, IL-8, IL-10, MIP-1β and IP-10); apoptotic (sFas and Fas-L); and angiogenic factors (VEGF and PDGFbb).

**Figure 3:**
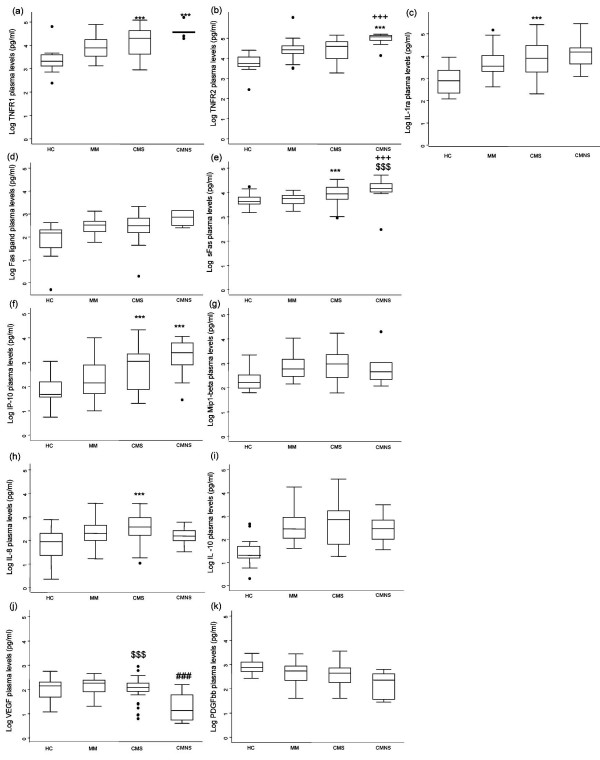
Biomarkers with significant differences between disease severity groups as determined by multivariate analyses. HC – healthy controls, MM – mild malaria, CMS – cerebral malaria survivors, CMNS – cerebral malaria non-survivors. Box plots represent medians and 25^th ^and 75^th ^percentiles. Bars mark the 10^th ^and 90^th ^percentiles, and outliers are plotted as points. MM versus CMS and CMNS: *** p < 0.05, $$$ p < 0.0045 bonferroni adjustment (alpha = 0.05/11), CMS versus CMNS: +++ p < 0.05, ### p < 0.0045 bonferroni adjustment (alpha = 0.05/11).

Having determined that these 11 factors showed important trends in different disease categories, the levels of these factors in all 133 samples (Figure 3) were further analyzed. The levels of sTNF-R1, sTNF-R2 and IL-1ra in plasma increased with disease severity (Figures 3a, 3b and 3c). The difference in the mean between CMS and each of the control groups (HC and MM) were significant for the three cytokine receptors (except for sTNF-R2 in the CMS vs. MM group) (Table 2). However, only sTNF-R1 and sTNF-R2 levels increased in the CMNS group as compared to CMS group, which was significantly different for sTNF-R2 (p < 0.05, Table 2). Expression of the apoptotic markers Fas-L and sFas also increased with disease severity (Figures 3d and 3e). However, only sFas showed significant differences across all disease groups, except HC vs. MM (Table 2). Fas-L levels of the HC were significantly different from the other groups (p < 0.05).

**Table 2:** Comparison of predicted means of least squares analyses of biomarkers by disease category

		CATEGORY					
		
BIOMARKER	OVERALL P-VALUE	HC VS. MM	HC VS. CMS	HC VS. CMNS	MM VS. CMS	MM VS. CMNS	CMS VS. CMNS
IL-1RA	0.0002	p < .05	p < .0045	p < .05	p < .05	NS	NS
IL-8	0.0003	p < .05	p < .0045	NS	p < .05	NS	NS
IL-10	<.0001	p < .0045	p < .0045	NS	NS	NS	NS
STNF-R1	0.0005	NS	p < .05	p < .0045	p < .05	p < .05	NS
STNF-R2	0.0002	p < .05	p < .0045	p < .0045	NS	p < .05	p < .05
VEGF	<.0001	NS	NS	p < .0045	NS	p < .0045	p < .0045
IP-10	0.0005	NS	p < .0045	p < .0045	p < .05	p < .05	NS
MIP-1β	0.0001	p < .05	p < .0045	NS	NS	NS	NS
PDGFbb	0.0056	NS	p < .05	p < .0045	NS	NS	NS
SFas	< .0001	NS	p < .05	p < .0045	p < .05	p < .0045	p < .05
Fas-L	0.0174	p < .05	p < .05	p < .05	NS	NS	NS

Significant differences in biomarkers in relation to disease severity determined by multivariate analyses (Comparison of Least Squares (Predicted) with Bonferonni correction) after modeling and controlling for covariates (age, sex, and parasitaemia).

Among the chemokines and cytokines evaluated, only IP-10 levels increased as disease severity increased (Figures 3f to 3i). The difference in the mean plasma level of IP-10 between HC and MM was not significant, but the differences between the CMS group and the control groups (HC and MM) were statistically significant (p < 0.0045 and p < 0.05, respectively, Table 2). Overall, IP-10 levels were highest in the CMNS group (Figure 3f) and was higher compared to the MM (p < 0.05), but not the CMS (p > 0.05), group (Table 2). Mean MIP-1β and IL-10 levels increased significantly in the CMS group in comparison to the HC group (p < 0.0045), but levels declined in the CMNS group when compared to CMS (Figures 3g and 3i), though not statistically significant (Table 2). Mean IL-8 levels were significantly higher in the CMS group compared to the HC and MM control groups, but not in the CMNS compared to the other groups (Figure 3h, Table 2).

In contrast to the inflammatory cytokines and apoptotic markers, levels of angiogenic growth factors (VEGF and PDGFbb) were lowest in severe disease. Among the selected factors, VEGF levels showed the largest difference between the CMS and CMNS groups (Figure 3j and Table 2). The decline in VEGF levels in CMNS were highly significant compared to the HC, MM and CMS groups (p < 0.0045). Similarly, PDGFbb levels (Figure 3k) were lowest in CMNS, and significant compared to HC (p < 0.0045). The differences in PDGFbb levels between CMNS and MM or CMS were not significant.

### Trend analysis using multinomial logistic regression

Since some factors showed important trend towards mortality associated with CM, we did additional analysis using a multinomial logistic regression to verify the statistical significance of these associations. As shown in Table 3, 10 out of 11 biomarkers are significantly associated with disease severity. Furthermore, biomarkers IL-1ra, IP-10, TNF-R1, TNF-R2, Fas-L, and sFas had increasing trend in odds ratio estimates for disease severity, including death. This suggests that for these specific biomarkers, as levels increase, odds of disease severity increase with odds of mortality being the highest. A protective effect against mortality was seen for VEGF levels as higher levels of VEGF decreased the risk of mortality (odds ratio CMNS: HC = 0.29). PDGFbb did not show any significant trend. While IL-8, IL-10 and MIP-1b are significantly associated with disease severity, the odds ratio estimates did not show a strict increasing trend as odds of CM to HC were higher than odds of CMNS to HC for increasing biomarker levels.

**Table 3:** Analysis of biomarkers for disease severity using multinomial logistic regression

BIOMARKER	COMPARISON OF DIFFERENT GROUPS	ODDS RATIO ESTIMATE	LOWER CONFIDENCE LIMIT	UPPER CONFIDENCE LIMIT	P-VALUE
log IL-1ra	CMNS:HC	4.159	2.257	7.666	<0.0001
	CM:HC	3.615	2.177	6.002	
	MM:HC	2.815	1.74	4.551	
log IL-8	CMNS:HC	1.596	0.922	2.762	0.0006
	CM:HC	2.542	1.636	3.952	
	MM:HC	1.815	1.214	2.712	
log IL-10	CMNS:HC	3.749	1.915	7.34	<0.0001
	CM:HC	4.38	2.364	8.115	
	MM:HC	4.21	2.278	7.777	
log TNF-R1	CMNS:HC	2.23	1.342	3.706	<0.0001
	CM:HC	1.541	1.259	1.887	
	MM:HC	1.406	1.176	1.682	
log TNF-R2	CMNS:HC	1.966	1.183	3.265	0.0003
	CM:HC	1.279	1.095	1.495	
	MM:HC	1.387	1.162	1.656	
log VEGF	CMNS:HC	0.299	0.149	0.6	<0.0001
	CM:HC	1.078	0.647	1.794	
	MM:HC	1.684	0.96	2.953	
log IP-10	CMNS:HC	2.724	1.71	4.337	<0.0001
	CM:HC	1.889	1.401	2.547	
	MM:HC	1.391	1.061	1.823	
log MIP-1β	CMNS:HC	2.901	1.419	5.932	0.0005
	CM:HC	3.78	2.038	7.013	
	MM:HC	3.244	1.763	5.968	
log PDGFbb	CMNS:HC	0.76	0.555	1.042	0.1863
	CM:HC	0.799	0.618	1.035	
	MM:HC	0.755	0.584	0.975	
log sFas	CMNS:HC	6.033	2.077	17.523	0.0021
	CM:HC	2.515	1.246	5.078	
	MM:HC	1.244	0.655	2.363	
log FasL	CMNS:HC	6.914	2.678	17.848	0.0001
	CM:HC	1.883	1.261	2.812	
	MM:HC	1.964	1.303	2.959	

HC (healthy control) was used as reference group. This analysis indicates the odds of risk (as indicated for each disease category compared to HC) for every one unit increase in log biomarker values. The p values represent the overall group differences. OR: odds ratio.

### Biomarker ratios and prognostic significance

**Figure 4:**
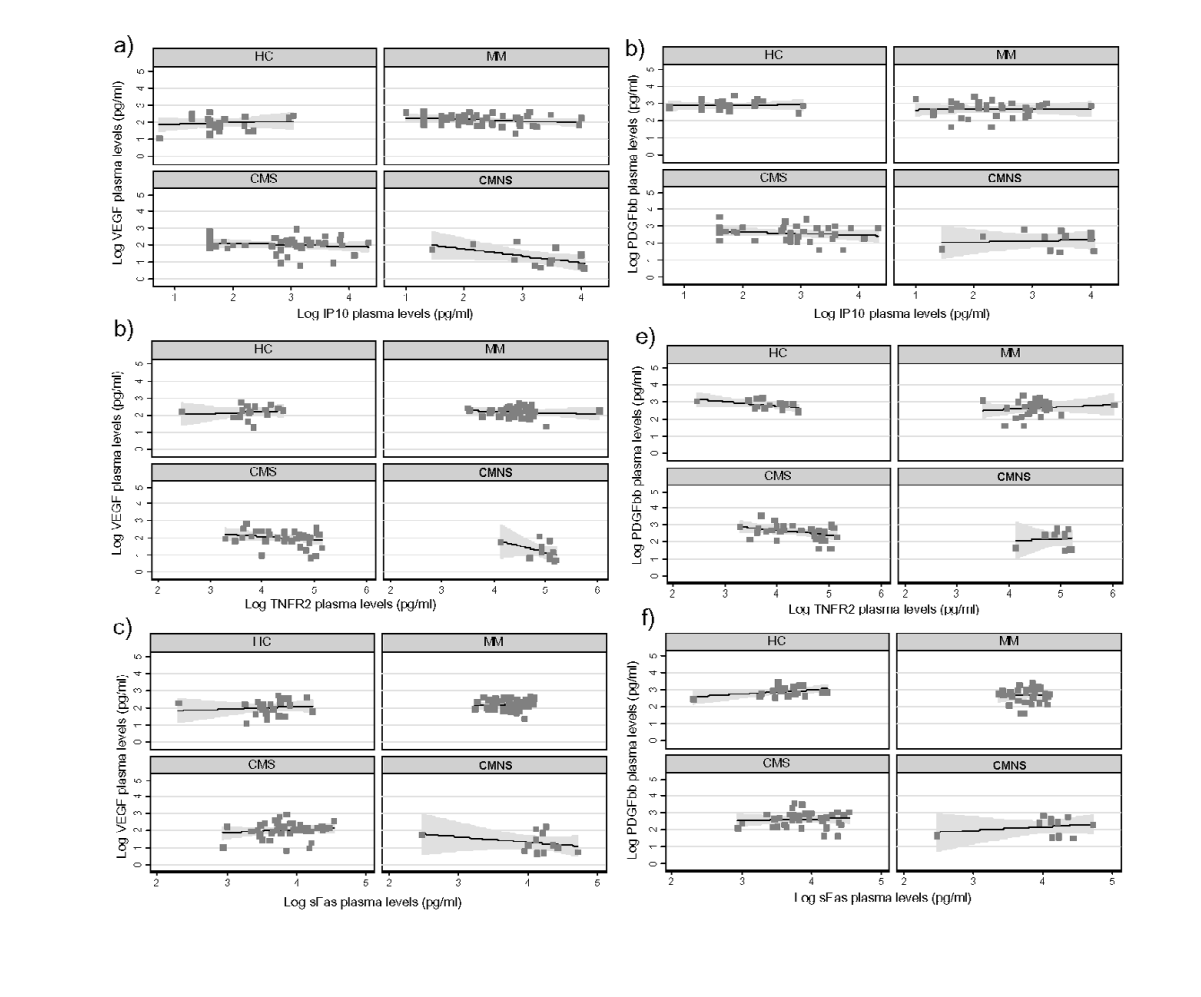
Relationship of the mean ratios of IP-10, sTNF-R2, and sFas to angiogenic protective factors VEGF and PDGF bb with disease severity. Line represents best fit with 95% confidence intervals in shaded regions.

The ratios of inflammatory markers to apoptotic and angiogenic factors were evaluated to determine differences between disease groups. Although the median IL-10:TNF-α ratio was lower in the CMS and CMNS groups as compared to the MM group, there were no significant differences in the mean ratio between the different groups (Table 4 and 5). However, both the mean and median of ratios of IP-10/VEGF, sTNF-R2/VEGF, and sFAS/VEGF increased as the disease severity increased, with the highest ratios in the CMNS group (Table 4). IP-10/PDGFbb, sTNF-R2/PDGFbb, and sFAS/PDGFbb ratios also increased with severity, but these ratios were about 10-fold less when compared to ratios with VEGF (Table 4). This difference is evident from the correlation coefficients shown in Figure 4. Most notably, the increase in these ratios between the CMNS group and other groups, including CMS, were highly significant (p < 0.0001, Table 5).

**Table 4:** Ratios of anti-inflammatory, angiostatic and apoptotic factors versus pro-inflammatory and angiogenic factors and relation to disease severity

	CATEGORY			
	
BIOMARKER RATIOS	HC	MM	CMS	CMNS
IL10:TNFα				
Mean	0.43	12.97	16.97	159.56
SEM	0.11	5.29	13.9	155.84
IP10:VEGF				
Mean	1.47	7.54	45.24	548.58
SEM	0.42	2.79	17.66	258.12
IP10:PDGFbb				
Mean	0.28	1.49	10.03	54.8
SEM	0.15	0.43	3.97	29.6
sTNFR2:VEGF				
Mean	34.7	469	1262	12945
SEM	17.98	168.2	461.45	4188
sTNFR2:PDGFbb				
Mean	11	160	286	1416
SEM	4.45	50.93	93.39	568
sFas:VEGF				
Mean	74	53.3	167	2090
SEM	16.37	9.3	47.07	813
sFas:PDGFbb				
Mean	8.78	23.6	50.8	212
SEM	1.75	5.21	17.59	88

The ratios of anti-inflammatory, angiostatic and apoptotic factors versus pro-inflammatory and angiogenic factors in different subject groups. SEM = standard error of the mean

**Table 5:** Predicted mean ratios of ANOVA analyses of CM prognostic biomarkers

		CATEGORY					
		
BIOMARKER RATIO	OVERALL P-VALUE	HC vs. MM	HC vs. CMS	HC vs. CMNS	MM vs. CMS	MM vs. CMNS	CMS vs. CMNS
IL10:TNFa	0.3096						
IP10:VEGF	<0.0001	NS	NS	<0.0001	NS	<0.0001	<0.0001
IP10:PDGFbb	0.0001	NS	NS	<0.0001	NS	<0.0001	0.001
STNFR2:VEGF	<0.0001	NS	NS	<0.0001	NS	<0.0001	<0.0001
STNFR2:PDGFbb	<0.0001	NS	NS	<0.0001	NS	<0.0001	<0.0001
sFas:VEGF	<0.0001	NS	NS	<0.0001	NS	<0.0001	<0.0001
sFas:PDGFbb	<0.0001	NS	NS	<0.0001	NS	<0.0001	<0.0001

Statistical comparison of predicted mean ratios of anti-inflammatory, angiostatic and apoptotic factors versus pro-inflammatory and angiogenic factors in different subject groups. P < 0.05 considered statistically significant; NS, not statistically significant.

## Discussion

Cerebral malaria is a major life-threatening complication of *Plasmodium falciparum *infection in humans. However, the mechanisms underlying fatal cerebral complications are still not fully understood. The present study was conducted to determine immunologically-relevant biological factors known to be associated with fatal CM and other severe forms of malaria in India. We examined disease associated inflammatory mediators, including cytokines, chemokines, apoptotic and angiogenic factors in sera of patients with HC, MM, CMS and CMNS. Due to the lack of concurrent studies of inflammatory, apoptotic and angiogenic biomarkers in appropriate time-matched controls from other diseases, the results are discussed in context. The study was conducted in an area of *P. falciparum *transmission where life-threatening complications of malaria occur.

The involvement of sTNF-R, sFas and IL-10 in the pathogenesis of CM and its associated mortality is consistent with previous reports. Mice deficient in TNF-R2 (TNF-R2-/-) or Fas ligand (Fas-/-) survive longer than wild type mice in experimental studies of CM, while TNF-R2-/- mice survive the longest in the absence of anti-malarial treatment [39]. The critical role of TNF-R2 in CM-associated death in experimental malaria has been well established [39-41]. In the absence of TNF-R2, mortality was eliminated in mice [39], while the restoration of TNF-R2 expression in brain tissue induced lethal CM [41]. Elevated levels of sTNF-R1 and sTNF-R2 in African children afflicted with CM have been reported, but no significant correlation with CM-associated mortality has been demonstrated [40,42]. This study has shown that plasma levels of sTNF-R2 are significantly higher in CMNS than in other groups. Since epidemiological and phenotypic differences exist between Indian and West African CM, further studies on the validity of these findings in South American, and other Asian settings may clarify these differences.

sFas and its ligand, Fas-L, are critical factors mediating malaria pathogenesis in both murine CM models and in human CM. Fas-deficient mice are protected against CM, while Fas and Fas-L expression increased in brain tissue of wild type mice with CM [43]. Fas and Fas-L interactions activate the classical extrinsic apoptotic pathway involving caspase cascades (caspase-8, -9, and -3) and activated caspase-3-positive apoptotic astrocytes have been found in mice affected by CM [8]. In a recent study in Gabon, Touré *et al *[44] demonstrated that although the factors inducing apoptosis have not been identified, apoptosis may play an important role in triggering some of the neurological manifestation associated with severe malaria. The present study has confirmed a robust positive correlation between sFas levels and CM fatality as was observed in postmortem CSF and serum in Ghanaian CM patients [22]. Thus, efforts are underway to test postmortem tissues and CSF from this Indian population in future investigations relating to CM mediated apoptosis and severity.

Mean IL-10 levels increased in the CMS group in comparison to the HC group but the levels declined in the CMNS group when compared to CMS. Interestingly, this decline coincided with a significant increase in IP-10 production as previously reported [22]. Interleukin-10 (IL-10) is a pleiotropic cytokine that inhibits the production of interferon-gamma (IFN-γ) by T helper 1 (Thl) cells and TNF secretion by macrophages. The protective role of IL-10 in CM severity has previously been demonstrated in murine CM models. In vivo neutralization of endogenous IL-10 in CM-resistant mice resulted in CM while increased production of IL-10 inhibited *P. berghei *ANKA antigen-specific IFN-γ and protected against CM in susceptible mice [45]. Recent studies have shown elevated levels of circulating IL-10 in patients with cerebral and severe malaria but less so in mild malaria. Thus, it seems that the decreased production of IL-10 in non survivors (CMNS) probably enhances production of IFN-γ and IP-10; an interferon inducible protein, to increase CM severity. Further mechanistic study is needed to determine the role of IL-10 in fatal outcomes of CM.

The study also revealed that VEGF may play a protective role against mortality associated with CM. VEGF is a vascular permeability factor with potent angiogenic and wound healing properties [46]. VEGF also interacts with PDGFbb and other factors such as thrombospondin-1 (TSP-1) and transforming growth factor beta (TGF-β) [47]. There is substantial evidence demonstrating the importance of VEGF in brain tissue repair and in CNS wound healing [48,49]. Exogenous administration of VEGF to injured brain tissues improved wound repair, functional response and decreased apoptosis in rats [50]. Decreased levels of serum VEGF and PDGFbb correlated with fatal outcome in CM. Interestingly, a decrease in levels of PDGFbb has been reported in CSF and serum of non survivors of CM [22]. Whether the decline in levels of VEGF or PDGFbb are causative agents of CM mortality or a specific phenotype of patients with susceptibility to fatal CM needs further evaluation. Based on this observation, it seems that significant decreases in VEGF may exacerbate neurological damage triggered by inflammatory- and/or parasite-derived factors, thus leading to death. It has been shown that neutralization of endogenous VEGF in the brains of injured rats results in decreased angiogenic activity and increased vascular and astrocyte degeneration, leading to larger wound cavities [48]. It is unclear why or how VEGF and PDGFbb production declines in the peripheral blood of CM patients. VEGF expression is negligible in the CNS under normal conditions, and it is activated after various types of neurological injuries [49]. In addition, elevated levels of VEGF-positive astrocytes and deposition of VEGF receptor-1 (VEGFR-1 or Flt-1) in Durck's granulomas were found in non-surviving CM patients [4]. Recently, elevated levels of VEGF in *P. falciparum*-infected primigravid mothers were reported [50]. Thus, it is evident that VEGF is involved in severe manifestations of malaria, although further studies will be required to fully understand its role in fatal outcomes of severe malaria. In a recent study involving Kenyan children, it was demonstrated that erythropoietin but not VEGF was associated with neurological sequelae [51]. It remains to be determined whether the differences between our study findings and the Kenyan study is due to differences in malaria epidemiology, immune status of the patients or host population genetics.

IP-10 (CXCL10) is a chemokine that is induced by IFN-γ, TNF-α, and other factors and has chemotactic activity for activated Th1 lymphocytes. There is growing evidence implicating this chemokine in both infectious and noninfectious causes of neuronal injury, dementia and inhibition of angiogenesis [52-54]. In this study, IP-10 levels were remarkably elevated as disease severity increases, with highest levels among non-survivors of CM (CMNS). These findings are consistent with recent studies on sera obtained from CM non-survivors in Ghana [22] as well as other reports implicating inflammatory and apoptotic mechanisms in the pathogenesis of CM [8-12].

This study confirmed an observed link between elevated levels of transcripts of genes implicating interferon-regulated processes and apoptosis in a murine CM model [55] and of elevated IP-10 in CM patients with poor prognosis [22]. It seems that TNF-α which is activated following the release of malaria antigens after schizont rupture, may activate production of IP-10 in brain capillaries and astrocytes [56,57]. IP-10 in concert with TNF-α can cause vascular injury resulting in breakdown in the blood brain barrier, which leads to accumulation of leukocytes that induce local hyper-inflammation. Significant down regulation of VEGF may further inhibit angiogenesis and regeneration of damaged blood capillaries. High systemic levels of IP-10, with its strong angiostatic properties, in CM patients may further inhibit angiogenesis and interfere with VEGF function. IP-10 also blocks VEGF interaction with its natural ligand by interacting with cell-surface glycosaminoglycans which provide accessory support for VEGF [58]. In viral meningitis, high levels of IP-10 in the cerebrospinal fluid (CSF) have been reported [54]. High levels of IP-10 have also been found in the CSF of patients with HIV-associated dementia correlated with neuropsychiatric impairment [59]. Furthermore, mouse studies have demonstrated that the HIV-1 virus-encoded protein gp120 directly activates astrocytes to produce IP-10 using a novel mechanism independent of IFN-α and the STAT-1 pathway of IP-10 induction [52]. In addition, elevated levels of IP-10, especially from astrcoytes, have been detected in Japanese encephalitis patients [60]. Based on these findings and those from other infectious disease models, it appears that IP-10 may play a more important role in CM-related brain encephalopathy/injury than previously thought. A very recent experimental CM study using knockout mice which lacked either IP-10, Mig or their receptor CXCR3, demonstrated that these molecules played a critical role in the CM associated death caused by *P. berghei *ANKA infection. It was further shown that IP-10 and Mig play a role in attracting CD8+T cells and NK cells to brains of infected mice and may be involved in causing death. In the infected mice, brain endothelial cells lining blood capillaries expressed both IP-10 and Mig while the neurons expressed IP-10 [61]. Collectively, these evidences suggest a prominent role for IP-10 in the pathogenesis of CM and associated death.

The inverse relationship between inflammatory factors and apoptotic and angiogenic markers were striking. In previous studies, the IL-10 (anti-inflammatory) to TNF-α (pro-inflammatory) ratio had been shown to be lower in severe malaria anaemia than in controls [62]. Interestingly, in this study sTNF-R2/VEGF, IP-10/VEGF and sFas/VEGF ratios were highest in the CMNS group; and were at least 10-fold higher as compared to the CMS group. This difference supports the hypothesis that the relative balance in inflammatory markers and apoptotic or angiogenic factors, especially VEGF, may predict risk of CM-associated mortality. These ratios could be used as prognostic markers in predicting fatal or severe outcomes associated with CM.

## Conclusion

This study has demonstrated that IP-10, sTNF-R2 and sFas (inflammation and apoptotic factors) are positively (risk factors for disease) correlated, while VEGF, an angiogenic and anti-apoptotic factor is negatively (protective) correlated with mortality associated with CM. The findings have provided new insights on the contribution of pathogen induced host factors to severity of CM and confirm recent findings that inflammatory chemokines such as IP-10 and factors involved in the extrinsic apoptotic pathway play a critical role in the pathogenesis of fatal CM. An important observation is the potential protective role of VEGF against fatal CM which may be worth investigating for development of strategies to improve treatment outcomes in CM patients. In addition, it has been demonstrated that the ratios of VEGF to IP-10, sTNF-R2, and sFas may indeed be used in predicting risk of developing fatal CM. Further analysis of these factors in patients dying of non CM-related causes and in CM patients from other geographic regions, will greatly improve current understanding of the pathogenic mechanisms involved in CM and enhance development of new prognostic markers and adjunctive therapies for improvement of treatment outcomes of CM.

## Abbreviations

CM: cerebral malaria; CSF: cerebrospinal fluid; CMS: cerebral malaria survivors; HC: healthy controls; CMNS: cerebral malaria non-survivor; MM: mild malarial; TNF: Tumor Necrosis Factor; IFN: Interferon; IL: Interleukin; FGF: fibroblast growth factor; G-CSF: granulocyte colony stimulating factor; GM-CSF: granulocyte-monocyte colony stimulating factor; IP-10: Interferon inducible protein 10; MCP: monocyte chemotactic protein; MIP: macrophage inflammatory protein; RANTES: regulated upon activation, normal T cell expressed and secreted; TGF: transforming growth factor; PDGF: platelet derived growth factor; VEGF: vascular endothelial growth factor; BBB: Blood-Brain Barrier; CNS: central nervous system; pRBC: parasitized red blood cell

## Competing interests

The authors declare that they have no competing interests.

## Authors' contributions

VJ, HBA, JET and NOW performed immunological experiments, proteomics analysis, data analysis and drafting of the manuscript. RMN participated in coordination of the study and technical training of field staff in India. SC participated in data analysis. PKJ, MPS, ACN and APD participated in the design and coordination of the study, and supervised patient recruitment and sample collection in India. JKS, VU and NS planned the study, participated in its design and coordination, supervision, interpretation of data and revised the manuscript for important intellectual content. All authors read and approved the final manuscript.
